# Food Additives, a Key Environmental Factor in the Development of IBD through Gut Dysbiosis

**DOI:** 10.3390/microorganisms10010167

**Published:** 2022-01-13

**Authors:** Pauline Raoul, Marco Cintoni, Marta Palombaro, Luisa Basso, Emanuele Rinninella, Antonio Gasbarrini, Maria Cristina Mele

**Affiliations:** 1 UOC di Nutrizione Clinica, Dipartimento di Scienze Mediche e Chirurgiche, Fondazione Policlinico Universitario A. Gemelli IRCCS, 00168 Rome, Italy; pauline.raoul1@gmail.com (P.R.); marco.cintoni@gmail.com (M.C.); luisa.basso@policlinicogemelli.it (L.B.); emanuele.rinninella@unicatt.it (E.R.); mariacristina.mele@unicatt.it (M.C.M.); 2 Dipartimento di Medicina e Chirurgia Traslazionale, Università Cattolica Del Sacro Cuore, 00168 Rome, Italy; antonio.gasbarrini@unicatt.it; 3 UOC di Medicina Interna e Gastroenterologia, Dipartimento di Scienze Mediche e Chirurgiche, Fondazione Policlinico Universitario A. Gemelli IRCCS, 00168 Rome, Italy

**Keywords:** IBD, food additives, gut microbiota, dysbiosis, artificial sweeteners, emulsifiers, colorants, Western diet, gut barrier, chronic inflammation

## Abstract

Diet is a key environmental factor in inflammatory bowel disease (IBD) and, at the same time, represents one of the most promising therapies for IBD. Our daily diet often contains food additives present in numerous processed foods and even in dietary supplements. Recently, researchers and national authorities have been paying much attention to their toxicity and effects on gut microbiota and health. This review aims to gather the latest data focusing on the potential role of food additives in the pathogenesis of IBDs through gut microbiota modulation. Some artificial emulsifiers and sweeteners can induce the dysbiosis associated with an alteration of the intestinal barrier, an activation of chronic inflammation, and abnormal immune response accelerating the onset of IBD. Even if most of these results are retrieved from in vivo and in vitro studies, many artificial food additives can represent a potential hidden driver of gut chronic inflammation through gut microbiota alterations, especially in a population with IBD predisposition. In this context, pending the confirmation of these results by large human studies, it would be advisable that IBD patients avoid the consumption of processed food containing artificial food additives and follow a personalized nutritional therapy prescribed by a clinical nutritionist.

## 1. Introduction

Globally, inflammatory bowel diseases (IBDs) affect more than 1 million individuals in the USA and 2.5 million in Europe, resulting in significant health and economic costs [1]. IBDs include ulcerative colitis (UC) and Crohn’s disease (CD). CD is a deeper transmural inflammatory condition that can occur in patches throughout the small intestine and the colon, while UC is characterized by mucosal inflammation limited to the colon [2]. Both these chronic inflammatory diseases of the gastrointestinal tract are mainly due to an abnormal mucosal immune response to intestinal microbiota to various environmental factors [3]. Although the etiology of these diseases seems to be multifactorial, the close relationship between dietary habits and IBD development has been demonstrated in recent decades [4]. Diet represents one of the key environmental factors impacting gut microbiota, directly influencing host homeostasis, gut inflammation, and immunological processes [5,6,7]. The gut microbiota constitutes a complex changing ecosystem composed of a huge number of species inhabiting the human gastrointestinal tract [8]. The bidirectional interactions between diet and gut microbiota might be involved in IBD as a therapeutic and preventive role or a causal risk factor.

In recent decades, the Westernization of lifestyle has led to the increasing consumption of (ultra-) processed foods. Processed foods often contain a mixture of non-nutritive ingredients, such as artificial food additives, used to improve or maintain the safety, freshness, taste, texture, or appearance of food products [9]. Artificial sweeteners such as aspartame, saccharin, sucralose, and acesulfame potassium offer a sweeter taste without calories. The replacement of sugars with these non-nutritive sweeteners reduces sugar and energy intake in many soft energy drinks, desserts, and snacks but can be an etiologic factor associated with the onset and progression of IBD [10]. Emulsifiers including lecithins, mono- and diglycerides, polysorbates, and carrageenan are commonly used in mayonnaise, salad dressing, and fruit juices to improve texture and shelf-life [11]. Other food additives, such as food colorants, are often used in pastries, sauces, ice-creams, and candies to contribute to food acceptance and selection by consumers. The use of food additives in food products is regulated by national authorities to avoid any microbiological and toxicological risks for the consumer. However, standardized methodologies to measure the actual food content of these diverse compounds and their potential effects on health are still limited [12].

Recently, the effects of artificial food additives on gut microbiota have received much attention in healthy patients as well as in patients affected with IBD [13]. Indeed, emerging evidence suggests interactions between artificial food additives and microbiota, which may affect host gut health. In preclinical studies, the high and prolonged consumption of food additives can be associated with the development of colitis and detrimental effects on gut homeostasis [14].

The present paper aims to gather the latest data, focusing on the potential role of different artificial food additives in the pathogenesis of IBDs through gut microbiota modulation. We present and discuss the current knowledge about the associations between different food additives, microbiota, and IBD onset and/or progression.

## 2. Methods

A systematic literature search was performed using Medline (via PubMed), Web of Science, and Scopus databases from the inception of the paper to October 2021. The search terms included “artificial food additives”, “artificial sweeteners”, “polyols”, “acesulfame potassium”, “aspartame”, “saccharin”, “sucralose”, “cyclamate”, “neotame”, “emulsifiers”, “carboxymethyl cellulose”, “polysorbate 80”, “food coloring agents”, “food preservatives”, “benzoic acid”, “sodium benzoate”, “titanium dioxide”, “sodium nitrite”, “food additive”, “inflammatory bowel disease”, “IBD”, “Crohn’s disease”, “ulcerative colitis”, “gut microbiota”, and “microbiome”. The search string for each database is described in Table S1 (Supplementary Materials). The relevant articles were identified, and hand-searching of eligible studies was carried out to check the reference lists and find additional references.

## 3. Interactions between Diet, Gut Microbiota, and IBD

### 3.1. Alterations of Gut Microbiota and Gut Barrier in IBD

In recent years, many studies have highlighted the role of gut microbiota in IBD development. In genetically predisposed individuals, intestinal dysbiosis induces an aberrant mucosal immune response and has a pivotal role in IBD pathogenesis [15]. Both CD and UC are characterized by an excessive T helper response to commensal microorganisms leading to impaired function of the gut barrier and increased intestinal permeability [16].

Recent advances in genetic sequencing and functional microbial analysis have provided means by which to characterize dysbiosis in patients with IBD. Many studies have found a generalized reduction in bacterial diversity (especially alpha diversity) as well as decreased representation of several specific taxa, particularly in anaerobic bacteria populations [17,18,19]. Essentially, the enteric microflora of IBD patients shows a decreased number of *Firmicutes* and *Bacteroidetes* and an increased number of *Enterobacteriaceae* [20]. Moreover, analysis of mucosal intestinal microbiota showed increased levels of *Streptococcus* in CD patients with active disease and sections of inflamed tissue in both CD and UC patients, as evidenced by lower levels of *Bifidobacterium* compared with non-inflamed tissue [21]. Recently Al-Bayati et al. conducted a longitudinal analysis of gut-associated bacterial microbiota in UC patients. Authors detected a significant reduction in *Faecalibacterium prausnitzii* (*F. prausnitzii*), *Prevotella,* and *Peptostreptococcus productus* (*P. productus*) compared with healthy control individuals [22]. *F. prausnitzii* is a major member of *Firmicutes* phylum, and it is one of the main butyrate producers found in the intestine [23]. Butyrate is a short-chain fatty acid (SCFA) with anti-inflammatory effects that alleviate oxidative stress in the epithelial layer of the gut [22]. *F. prausnitzii* deficiency has been reported and studied in patients with CD or UC [21,22,23]. Interestingly, deficient colonization of *F.prausnitzii* was observed in UC patients during active disease, while the recovery of the *F. prausnitzii* population was associated with maintenance of remission [24,25,26]. Interestingly, a recent study evaluated dysbiosis in IBD subtypes by calculating alpha- and beta-diversity and dysbiosis scores [27], showing evidence of dysbiosis in IBD in CD compared to healthy controls, following the trend of previous studies based on the 16S r ribonucleic acid (RNA) approach [28,29,30]. Moreover, the dysbiosis score in CD seems to be higher compared with UC [27,29]. Specifically, in CD, an increase in some species such as *Escherichia coli*, *Ruminococcus gnavus*, and *Clostridium clostridioforme* and the depletion of *F. prausnitzii* were observed compared to UC and healthy controls [27].

In parallel, the use of antibiotics in IBD management also increases intestinal dysbiosis, reducing the abundance of beneficial bacteria (such as *Bifidobacterium*, *Lactobacillus*, *Bacteroidetes*, and *Firmicutes*) and allowing the increase in pathogenic bacteria (such as *E. coli*) [16]. Thus, the presence of dysbiosis in CD and UC is evident, although it remains unclear if dysbiosis is a driver of inflammation or a result of inflammation.

Dysbiosis affects gut barrier permeability and intestinal homeostasis, representing a continuous balance between immune reaction and toleration among host immune response and luminal flora [15]. The gut barrier plays a fundamental role, and a healthy mucous layer is essential to keep the balance. Immunoglobulin (Ig)A, secreted by enterocytes, is a very important messenger, preventing inflammatory response to gut commensal and blocking the invasion of non-commensal pathogens. In UC, the lack of the mucous layer is associated with impaired operativity of IgA [15].

An important group of protective peptides in the intestinal mucus layer is antimicrobial peptides (AMPs), in particular defensins [15]. Defensins are innate immune system antibiotics able to bind and destroy the membrane of a wide range of Gram-positive and Gram-negative bacteria as well as yeast [31]. Defensins production occurs when microbial products stimulate a family of cytoplasmic proteins, called nucleotide-binding oligomerization domain (NOD)-like receptor (NLR) [32]. In CD a mutation of NLRs (NOD2 or NLRP3) determines an impaired production of defensins, resulting in microbial invasion and epithelium inflammation [33,34]. Other relevant components of intestinal mucosal epithelium are goblet cells, which are mucosal cells that synthesize and secrete mucus [35]. Their role is essential in maintaining intestinal homeostasis because they form a physical barrier against luminal toxins and protect the integrity of epithelium, supporting hydration [15]. In UC, goblet cell number and size drastically decrease, owing to downregulation of differentiation, and this is associated with a thinner or absent mucus layer and thus inflammation [36]. Innate and adaptive inflammatory cells infiltrating the lamina propria can produce pro-inflammatory cytokines such as interferon (IFN)-γ, interleukin (IL)-17, tumor necrosis factor (TNF)-α, or IL-1β, exacerbating the inflammatory process and causing epithelial damage and intestinal symptoms.

### 3.2. Role of Diet in IBD

#### 3.2.1. Diet, a Therapeutic Role for IBD

Evidence that diet is an important determinant in the course of IBD is proved by the fact that exclusive enteral nutrition (EEN) is effective in inducing clinical and endoscopic remission [37]. EEN is currently used as first-line therapy in the pediatric population and showed promising results in adults with a good response in terms of both symptoms and inflammatory markers [38]. The mechanism through which EEN carries out its beneficial action seems to be related to different mechanisms: bowel rest, effects of anti-inflammatory nutrients included in enteral formulae, and reduced exposure to dietary risk factors [39]. Given the demonstrated relevance of diet, several attempts have been made to find the best alimentary regimen to improve symptoms and limit relapses in IBD patients [40,41]. An example is the CD treatment-with-eating diet (CD-TREAT). This is a personalized food-based diet with a composition as similar as possible to that directed by EEN, which is based on the exclusion of certain dietary components (gluten, lactose, and alcohol) and the matching of others (macronutrients, vitamins, minerals, and fiber) [42]. CD-TREAT showed similar benefits as EEN and has the advantage of being more palatable and better tolerated [42]. Chiba et al. successfully tested a semi-vegetarian diet (SVD), structured as a lacto-ovo vegetarian diet with the addition of fish once a week and meat once every 2 weeks for CD patients. SVD was effective in inducing remission and preventing relapse in 2 years of follow-up [43].

The Mediterranean diet can be also recommended for IBD patients [44]. The strength of this diet is essentially in its content of extra-virgin olive oil, which is a precious source of polyphenols. These compounds help prevent oxidative damage in colon cells and improve symptoms of chronic inflammation by inhibiting arachidonic acid and nuclear factor (NF)-κB signaling pathways [41]. Moreover, flavonoids seem to be implicated in the maintenance of the thigh junctions and are thus important for intestinal barrier integrity [45]. Several studies demonstrated that phenolic compounds can positively influence the gut microbiota, resulting in a greater abundance of beneficial microbes [46,47,48].

The low-FODMAP diet (fermentable oligosaccharides, disaccharides, Monosaccharides, and polyols) has also proven its efficacy in ameliorating gastrointestinal symptoms. This alternative diet has gained popularity among physicians, especially for its use as a treatment option in IBD [5]. However, its long-term impact on the microbiota appeared disadvantageous with a general decrease in butyrate-producing bacteria [49]. Moreover, a low-FODMAP diet can lead to a reduction in potential prebiotics (fructo-oligosaccharides and galacto-oligosaccharides), thus leading to a reduction in beneficial bacteria and fermentative effects [50].

The gluten-free diet is sometimes prescribed for IBD patients to alleviate symptoms. Both IBD and celiac disease contribute to the dysregulation of innate and adaptive immune responses, leading to chronic inflammation. There is a strong association between celiac disease and colitis [51]. Interestingly, a recent study showed that nearly one-third of IBD patients report a diagnosis of non-celiac gluten sensitivity, and many follow a gluten-free diet [51]. However, similarly to the low-FODMAP diet, it is associated with unfavorable changes in microbiota composition [49]. Indeed, a decrease in healthy bacteria such as *Bifidobacterium* and *Lactobacillus* has been demonstrated, leading to a diminution of SCFAs production and their beneficial metabolic and host immunity effects [52]. Moreover, the increase in detrimental species such as *Enterococcus*, *Staphylococcus*, *Salmonella*, *Shigella*, and *Klebsiella* can influence the microbial profiles and impact the intestinal mucosa in IBD patients [53,54].

Despite the numerous attempts to define a better nutritional strategy, there is still not a consensus on the proper diet, since the evidence is still unclear [55].

#### 3.2.2. Western Diet, a Causal Role in the Onset and Progression of IBD

The highest incidence of IBD is in North America and Europe, while in Africa, Asia, and South America its incidence and prevalence are lower [56]. Interestingly, several epidemiological studies evidenced that IBD incidence is progressively rising in developing countries in association with the introduction of Western lifestyles and eating habits [57]. Moreover, people emigrating from low-prevalence regions to high-prevalence countries have an increased risk of developing IBD, and this is especially true for children [58]. All this evidence makes it clear that environmental factors such as Western dietary patterns negatively impact IBD risk [55].

The Western diet is characterized by elevated total energy consumption, a high intake of animal fat and protein, an excess of sugar and salt, a low fiber content, and high consumption of processed foods [59]. It is associated with unfavorable changes in gut microbiota and impaired gut homeostasis [49]. The microbial diversity decreased in mice fed with a high-fat/high-sugar diet with a specific reduction in barrier -protective bacteria and an increase in opportunistic pathogens, especially adherent-invasive *E. coli* (AIEC) [60]. Moreover, mice showed decreased mucus layer thickness, increased intestinal permeability, and increased TNFα secretion [60].

The Western diet is characterized by a low intake of dietary fiber found in fruit and vegetables. Dietary fiber seems to positively influence the intestinal microbiome. Dietary fiber remains relatively intact in the human colon, where it is fermented by specific bacteria with the production of SCFAs, lactate, and gas [61]. These products of fermentation exert anti-inflammatory effects and promote the proliferation of benefits as *Bifidobacteria* and *Lactobacilli* [62]. A diet rich in fruit and vegetables, rich in n-3 fatty acids, and low in n-6 fatty acids is associated with a decreased risk of developing CD and UC, and it is, therefore, recommended [63].

Among other diet components, in particular, red meat and processed meat are positively associated with CD and UC and may aggravate the course of the disease [64]. Animal proteins are strongly associated with IBD risk [41,65]. Furthermore, high amounts of saturated fatty acids (SFAs) in animal models determine changes in bile acid composition, and this stimulates the growth of sulfate-reducing bacteria, which induce gut inflammation and damage the intestinal membrane [66]. In a large prospective study in French middle-aged women, high total protein intake, especially of animal origin, was related to IBD risk [40]. Regarding the source of animal protein, meat especially seemed to be implicated [40]. Indeed, meat is a source of *N*-6 polyunsaturated arachidonic acid (AA), which is the precursor of several pro-inflammatory molecules such as prostaglandins, leukotrienes, and thromboxane [39]. Observations suggest that *N*-6 polyunsaturated fatty acid may contribute to a predisposition to UC, while *N*-3 polyunsaturated fatty acid may be protective [39]. A high N-6-to-N-3 PUFA ratio has been identified in patients with CD and has been associated with disease activity [20]. Additionally, meat is the main source of haem, which, together with its product, iron, may promote inflammation through the generation of reactive oxygen species [39]. Moreover, protein fermentation within the colonic microbiota produces ammonia and hydrogen sulfide (H_2_S), which affects the integrity of the mucus layer and displays a pro-inflammatory effect [67].

Recently, a significant positive association between a higher intake of ultra-processed food and risk of IBD was shown in a prospective study of more than 115,000 adults [68]. Ultra-processed foods are composed of not only sugar and fat but also a series of other non-nutritive substances called food additives. Certain food additives such as probiotics and prebiotics have beneficial health effects since they are used to directly or indirectly influence gut microbiota to improve the health condition of the host [69]. Many other food additives—such as emulsifiers, preservatives, artificial sweeteners and flavorings, and coloring agents—are used to increase palatability, modify texture, and prolong shelf life. These kinds of food additives can have potential toxicity and undesired side effects on health. Toxicity studies are carried out following a series of guidelines, including clinical and biochemical analyses [70]. Based on these results, food additives are allowed to be used at an estimated innocuous and safe amount called the acceptable daily intake (ADI).

Herein, we present how, more recently, these substances have been associated with bacterial dysbiosis, microbiota colonization, and metabolism, thereby modulating the activity of inflammatory and immune systems.

## 4. Food Additives, Gut Microbiota, and IBD

### 4.1. Artificial Sweeteners, Gut Microbiota, and IBD

**Figure 1:**
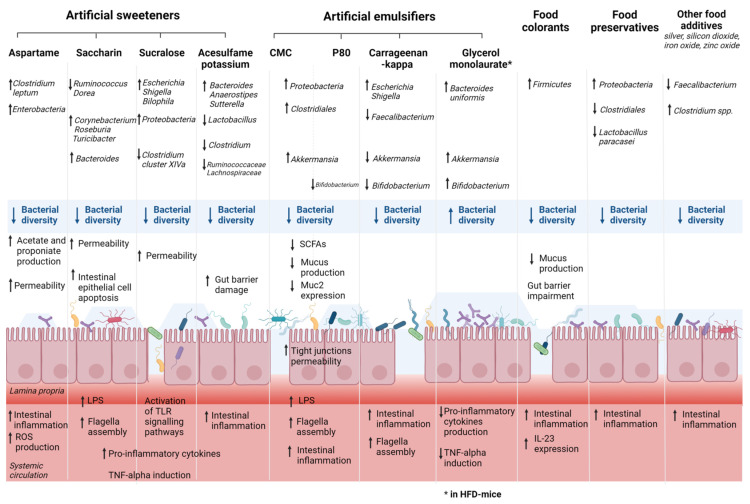
Effects of exposure of some artificial food additives on gut microbiota and gut barrier. These main findings are based on in vivo studies detailed within the manuscript. Abbreviations: CMC, carboxymethyl cellulose; HFD, high-fat diet; IL, interleukin; LPS, lipopolysaccharide; P80, polysorbate 80; ROS, reactive oxygen species; SCFA, short-chain fatty acid; TLR, Toll-like receptor; TNF, tumor necrosis factor.

Artificial sweeteners, also called non-nutritive sweeteners, are commonly found in many types of foods and beverages and are often recommended for bodyweight reduction for patients with type two diabetes mellitus and glucose intolerance [71]. The Food and Drug Administration (FDA) and the European Food Safety Authority (EFSA) approved the use of several artificial sweeteners such as aspartame (E951), saccharin (E954), sucralose (E955), acesulfame potassium (E950), and neotame (E961). However, a growing number of animal studies have produced controversial results on the effect of these artificial sweeteners on gut bacteria, gut barrier, and immune functions, which can negatively affect individuals with or susceptible to chronic inflammatory conditions such as IBD. Recent RCTs have been also published, although they remain scarce in terms of sample size. Table 1 summarized the results of recent animal and human studies assessing the effects of common artificial sweeteners on gut microbiota composition, the gut barrier, and inflammatory system. Figure 1 illustrates the main results of preclinical studies assessing the effects of the exposure of some artificial sweeteners (aspartame, saccharin, sucralose, and acesulfame potassium) on gut microbiota.

Recent mice model studies demonstrated a reduction in gut bacterial diversity with an alteration of bacterial communities leading to dysbiosis after consumption of acesulfame potassium [72,73], sucralose [73,74,75,76,77], sucrose [76], or Splenda^®^ [78] as well as aspartame [79] and neotame [80]. Indeed, the consumption of sucralose, but not acesulfame potassium, can reduce the relative amount of *Clostridium cluster XIVa* [73]. Other studies assessed an increase in *Bacteroides*, significant changes in *Anaerostipes* and *Sutterella* abundances, and a decrease in abundance of *Lactobacillus* [81], *Clostridium* [72,81], *Lachnospiraceae* [72], *Ruminoccocaceae* [72] in acesulfame potassium-treated male mice.

Sucralose consumption can lead to intestinal microbial changes [76,77] with an increase in the abundance of *E. Coli/Shigella* and *Bilophila* in mice [82]. Aspartame can also alter the microbial composition by reducing the total bacteria abundance and increasing the abundance of *Enterobacteriaceae* and *Clostridium leptum* [79]. Bian et al. [83] demonstrated an association between saccharin-induced liver inflammation and changes in the intestinal microbiota such as *Ruminococcus*, *Adlercreutzia*, *Dorea*, *Corynebacterium*, *Roseburia*, and *Turicibacter*. The impact of consumption of the commercial sweetener (Splenda^®^) containing sucralose and maltodextrin (MDX) on both the severity of CD-like ileitis and the intestinal microbiome alterations was studied in mice [74]. Splenda^®^ can promote dysbiosis with the expansion of *Proteobacteria* and *E. coli* and increased myeloperoxidase. Interestingly, although Splenda^®^ may promote microbiome alterations in CD-prone and healthy hosts, myeloperoxidase levels in healthy mice did not increase compared with CD-prone mice [74]. Thus, a hypothesis is that the consumption of sucralose/MDX-containing foods might exacerbate myeloperoxidase intestinal reactivity only in individuals with a pro-inflammatory predisposition such as CD. On the contrary, stevia can increase gut microbial diversity [76], especially Shannon alpha diversity [82].

As regards human studies, the number of RCTs with healthy volunteers is growing. One recent study observed the fecal samples of healthy volunteers before and after consumption of aspartame and sucralose [84], and another studied fecal samples after treatment of saccharin [85]. In both studies, minimal effects were found in terms of gut microbiota compositional variations and SCFAs production [84,85]. In addition, it is important to note that in these studies, the daily dose of artificial sweeteners was less than 20% of the ADI for aspartame and sucralose, and the duration of the treatment did not exceed ten weeks. SCFAs, primarily acetate, propionate, and butyrate, are the products of the bacterial fermentation of dietary fiber in the colon. SCFAs act as anti-inflammatory metabolites in the gut, particularly via the regulation of T-regulatory cells. Moreover, SCFAs are an important energy source for intestinal epithelial cells and are known to strengthen the gut barrier function. In IBD, SCFAs are typically reduced in gut mucosa and feces of patients with IBD. Several studies found that sucralose increased the number of SCFA-related genes, especially in a diet rich in saturated fats [86]. Additionally, aspartame consumption can increase the levels of circulating SCFAs, particularly propionate [79,82] and acetate [82], while neotame intake can decrease the expression of butyrate synthetic genes [80].

These microbial changes are associated with modification of the pathophysiology of the intestinal barrier. Increased permeability was observed in various mice model studies after consumption of acesulfame potassium [72] or sucralose [75,77,87]. Induced intestinal injury with enhanced lymphocyte migration to intestinal mucosa was observed in mice consuming acesulfame potassium [72]. Moreover, the expression of glucagon-like peptide (GLP)-1 and 2 receptors was significantly reduced in acesulfame potassium-treated animals. GLP signaling modulates the epithelial barrier function by tightening intercellular junctions [72]. Santos et al. studied the effect of acesulfame potassium, sucralose, aspartame, and saccharin in vitro on intestinal barrier functions and found that only saccharin increased permeability [88]. A recent study found that at high concentrations, aspartame and saccharin can induce apoptosis and cell death in intestinal epithelial cells, while at low concentrations, sucralose and aspartame increased epithelial barrier permeability and down-regulated claudin 3 at the cell surface [89,90]. Thus, various artificial sweeteners such as acesulfame potassium and aspartame can exacerbate the impairment of the intestinal mucus layer observed in CD with impaired production of defensins, resulting in microbial invasion and epithelium inflammation and UC patients with impaired operativity of IgA [88].

An increased expression of pro-inflammatory cytokines such as IL1b, IL6, IL8, and IL12 was observed in various animal studies assessing the impact of acesulfame potassium [72], sucralose [76,77,86,91,92], sucrose [76], and Splenda^®^ [78]. Consequently, in predisposed IBD patients, the consumption of these food additives can aggravate intestinal inflammation with an enhancement of the production of IL-1β associated with IBD pathogenesis, as we previously mentioned.

In addition, the expression of bacterial pro-inflammatory mediators such as lipopolysaccharide (LPS) and flagella protein synthesis is exacerbated after consumption of sucralose, steviol, sucrose [91], or saccharin [83]. LPS activates pathways that are directly involved in the progression of CD and UC, particularly the NF-κB pathway, which increases levels of IL-1, IL-6, and TNFα [93]. On the other hand, a reduction in anti-inflammatory cytokine IL10 was found in mice fed sucralose or stevia [92]. Interestingly, mice fed sucralose with dextran sulfate sodium (DSS) experienced an exacerbation of the severity of the colitis [75,77], during which the TLR5-MyD88-NF-κB signaling pathway was most likely activated [77]. TLRs are key sensors in the gut that recognize abnormal intestinal microbes and induce an immune response and IBD. The abnormal TLR signaling may trigger disease-related inflammation [94].

### 4.2. Emulsifiers, Gut Microbiota, and IBD

Emulsifiers are added in a wide variety of processed foods to enhance texture and extend shelf-life, but these substances, particularly synthetic emulsifiers, can impact gut microbiota and promote chronic intestinal inflammation. Table 2 details the results of studies showing the effect of the most common emulsifiers on gut microbiota in animal and human models. Figure 1 illustrates the main findings of studies assessing the effects of the exposure of some artificial emulsifiers on gut microbiota. The majority of studies are animal model studies, and there is a crucial need for randomized controlled trials to confirm these results in humans with an adequate intake of food additives.

In mice, consumption of emulsifiers promotes an alteration of microbial composition in the gut. Indeed, the most studied substances are carboxymethyl cellulose (P80) and carboxymethyl cellulose (CMC). Several studies demonstrated that their consumption can decrease microbial diversity [95,96,97,98,99] and induce gut bacterial variations [100] such as a growth of *Gammaproteobacteria*, known for promoting mucosa-associated inflammation [95,96,101,102], an increase in *Bacteroidales* [100], *Bacteroidetes* [100], and a decrease in *Clostridiales* [97,100] and *Lactobacillus* [100]. Additionally, the maternal consumption of P80 in mice can induce dysbiosis in offspring with an increase in *Proteobacteria*, *Helicobactraceae*, *Campylobacterales*, and *Desulfovibrionales* [103]. Recently, Naimi et al. studied 20 dietary emulsifiers using a MiniBioReactor Array Model, demonstrating that not only P80 and CMC can cause dysbiosis but also carrageenans, gums, and sunflower lecithin. A decrease in *Clostridiales* such as the *Faecalibacterium* genus was found after the intake of P80, iota carrageenan, mono-diglycerides, while an increase in the *Bacteriodales* order may be associated with the consumption of kappa carrageenan, lambda carrageenan, and glyceryl stearate [97]. Gerasimidis et al. confirmed these findings in an in vitro human microbiota study demonstrating an increased abundance of *Escherichia coli/Shigella* after carrageenan kappa consumption and inhibition of *Bifidobacterium* by P80 and carrageenan-kappa [82]. As previously mentioned, similar microbial variations in CD and UC patients such as a depletion in abundances of *Faecalibacterium* and *Bifidobacterium* species are found in various studies [27] as well as an increase in levels of *Escherichia coli* [20,27]. Therefore, we can hypothesize that certain emulsifiers including P80 and carrageenan-kappa can exacerbate some specific microbial variations associated with CD or UC pathogenesis. Carrageenan can also decrease levels of the anti-inflammatory bacterium *Akkermansia muciniphila* [104]. Compared with healthy controls, the colonization rate and relative abundance of *A. muciniphila* in IBD patients were significantly reduced, which was more evident in UC [105,106]. Thus, carrageenan consumption can aggravate the reduction in this probiotic gut bacterium, as already demonstrated, especially in UC.

All these microbial changes have been associated with alterations of the gut barrier and intestinal permeability. Several studies demonstrated increased tight junction permeability, increased bacterial translocation, and decreased mucus production in mice fed with CMC leading to increased inflammation [56,95,100,107]. P80 consumption by mice appears to have similar effects to CMC [95,100,108] on the gut barrier with a reduction in Muc2 RNA expression and reduced mucus thickness in the intestinal epithelium. Maternal P80 consumption leads to gut barrier disruption in offspring and exacerbation of DSS-induced colitis in offspring adulthood [103]. Moreover, Fukuhashi et al. also found 1% P80 can induce small intestine vulnerability to indomethacin-induced lesions [96].

However, interestingly, some emulsifiers can have a positive impact on gut microbiota composition. Glycerol-monolaurate can promote dysbiosis in mice fed with a low-dose supplementation in a low-fat diet but can improve high-fat diet (HFD)-induced gut microbiota dysbiosis, increasing abundances in *Bacteroides uniformis*, *Akkermansia*, *Bifidobacterium*, and *Lactobacillus* and reducing levels of *E. coli*, *Lactococcus* [109,110]. Other emulsifiers such as rapeseed lecithin and soy lecithin can increase levels of butyrate production bacteria such as *Clostridium leptum* [111]. In predisposed or diagnosed IBD patients, the consumption of food products containing glycerol-monolaurate cannot aggravate microbial variations associated with IBD such as increased levels of *E. coli* and a decreased abundance of *Bifidobacterium*.

The loss of barrier integrity can lead to the translocation of bacterial antigens promoting the colonization of intestinal bacterial pathogens, which cause inflammation. Several studies assessed this downstream effect in mice consuming emulsifiers such as P80 or CMC. Indeed, increased levels of LPS and flagellin as indirect measures of gut permeability were observed leading to an increase in gut inflammation [96,99,101,102,104,107,108,110]. Naimi et al. [97] assessed the effect of more than 20 emulsifiers on human microbiota maintained ex vivo in the MiniBioReactor Array Model. MDX, xantham gum, sorbitol monostearate, glyceryl stearate can increase LPS levels, and all carrageenans increased levels of flagellin. Moreover, carrageenans, gums, P80, and CMC increased gut inflammation. Laudisi et al. [14] focused on MDX, showing that this food additive can promote reticulum stress-driven mucus reduction and can exacerbate intestinal inflammation, especially in predisposed and diagnosed IBD patients.

Inflammation itself can also impact microbiota composition, exacerbating dysbiosis and potential colitis, promoting the development of chronic inflammatory diseases such as IBD. Recently, Chassaing et al. [98] performed a double-blind controlled feeding study of CMC (15 g per person per day representing a total amount of emulsifiers by persons whose diets largely comprised highly processed foods) in healthy adults. Compared with controls, CMC reduces bacterial gut diversity and beneficial metabolomes such as SCFAs. Thus, most emulsifiers can perturb the host–microbiota relationship promoting inflammation and development or exacerbation of chronic IBDs.

**Table 2:** Effects of emulsifiers on gut microbiota and immune/inflammatory system in animal and human models.

First Author, Year	Food Additives	Model	Main Results		
			Impact on Microbiota Composition	Impact on the Gut Barrier and Intestinal Permeability	Impact on the Immune and Inflammatory System
Chassaing, 2015 [95]	CMC P80	Mice	Reduction in microbial diversity Enrichment in Verrucomicrobia phyla, especially *Akkermansia muciniphila* Enriched mucosa-associated inflammation-promoting Proteobacteria	Decreased mucin production	Increased inflammation Increased levels of bioactive LPS and flagellin in WT, IL10−/−, and TLR5−/− mice Increased fecal LCN2 expression
Chassaing, 2017 [99]	CMC P80	Mice M-SHIME	In mice: CMC and P80 do not impact intestinal microbiota or host in mice harboring a pathobiont-free microbiota Using the M-SHIME: P80 and CMC directly alter the microbiota		Increased inflammation Increased levels of bioactive flagellin
Chassaing, 2021 [98]	CMC	Human RCT	Reduced gut bacterial diversity	Reduced SCFAs	
Furuhashi, 2021 [96]	P80	Mice	Increased *Gammaproteobacteria* abundance Decreased α-diversity in the small intestine No decrease in α-diversity in the colon Increase in sulfide-producing bacteria *Proteus* spp.	Exacerbation of the indomethacin-induced small-intestinal lesions Direct enhancement of the motility of specific flagellated microbiota	Increase in IL-1β expression
Gerasimidis, 2019 [82]	P80 Carrageenan-kappa	In vitro human microbiota	Carrageenan-kappa: Increased abundance of *Escherichia/Shigella* P-80: Decreased levels of *Faecalibacterium* and Subdoligranulum Both: Inhibition of growth of *Bifidobacterium*	P80: Increased propionic acid levels	
Jin, 2021 [103]	Maternal P80	Mice	Induction of dysbiosis in offspring with an increase in Proteobacteria, *Helicobacteraceae*, *Campylobacterales*, and *Desulfovibrionales*	Gut barrier disruption	Aggravation of the structural disorder of intestinal crypts Increased inflammation Exacerbation of DSS-induced colitis in offspring adulthood
Miclotte, 2020 [112]	A total of 5 emulsifiers: CMC, P80, soy lecithin, sophorolipids, rhamnolipids	In vitro human microbiota	Sophorolipids and rhamnolipids: Increased abundance in potentially pathogenic genera-like *Escherichia/Shigella* and Fusobacterium, a decreased abundance of beneficial *Bacteroidetes* and *Barnesiella* For all: Decline in intact microbial cell counts	Sophorolipids and rhamnolipids: Increase in flagellar assembly and general motility Decreased SFCAs production (especially butyrate and propionate)	
Naimi, 2021 [97]	A total of 20 dietary emulsifiers (1)	Human microbiota maintained ex vivo in the MiniBioReactor Array Model	P80, CMC, carrageenans, gums, and sunflower lecithin: Induction of dysbiosis P80, iota carrageenan, and mono-diglycerides: Decrease in *Clostridiales* order, especially *Faecalibacterium* genus Kappa carrageenan, lambda carrageenan, and glyceryl stearate: Increase in *Bacteroidales* order P80, CMC, carrageenans, and gums Decrease in microbial bacterial density Soy lecithin and glyceryl oleate No impact on gut microbiota Sorbitan monostearate and glyceryl stearate Increased microbial density		Maltodextrin, xantham gum, sorbitan monostearate, and glyceryl stearate: Increased LPS levels All carrageenans: Increased levels of flagellin Carrageenans, gums, P80, and CMC: Increased inflammation
Robert, 2021 [111]	Rapeseed lecithin Soy lecithin	Mice	Increased levels of *Clostridium leptum* (butyrate production bacteria)		Beneficial anti-inflammatory effects
Rousta, 2021 [101]	CMC P80	Mice	CMC: no bacterial compositional changes but decrease in *Uroviricota*, driven by changes in the *Caudoviricetes* bacteriophage class. P80: selectively expanding *Gammaproteobacteria*		Increased fecal LCN2 levels Increased colonic inflammatory cytokine expression Exacerbated colitis in ex-germ free IL10−/− mice colonized with fecal microbiota from patients with active IBD to a greater degree than does P80
Sandall, 2020 [113]	CMC P80 soy lecithin gum arabic	Mice			Increased inflammation
Shang, 2017 [104]	Carrageenan	Mice	Decrease in the abundance of *Akkermansia muciniphila*		Increased inflammation Induction of colitis
Singh, 2016 [108]	P80	Mice	Increase in Gram-positive bacteria	Reduced Muc2 RNA expression Reduced mucus thickness in the intestinal epithelium Increased gut permeability	Increased inflammation Increased level of LPS Increased level of flagellin Increased LCN2 expression
Swidsinki, 2009 [107]	CMC	Mice		Increased tight junction permeability Increased bacterial translocation	Increased inflammation
Viennois, 2017 [102]	CMC P80	Mice	CMC and P80: Significant reduction in microbiota diversity Increase in *Bacteroidales* Decrease in Clostridiales orders by CMC or P80 consumption Decrease in Firmicutes, such as Lactobacillus Increase in *Bacteroidetes*		Increased inflammation
Viennois, 2020 [100]	CMC P80	Mice	Alteration of intestinal microbiota composition	Increased motility and ability to adhere to intestinal epithelial cells Increased microbiota encroachment	Increased chronic inflammation
Zhao, 2019 [109]	GML	Mice	Improvement of HFD-induced gut microbiota dysbiosis Increase in levels of *Bacteroides*, *Akkermansia*, *Bifidobacterium*, and *Lactobacillus* Reduction in *E. Coli*, *Lactococcus*, and *Flexispira*		Reduced serum proinflammatory cytokines Reduced production of TNF-alpha Attenuation of LPS load
Zhao, 2020 [110]	GML	Mice	Modulation of HFD-induced gut microbiota dysbiosis Increased abundance of *Bifidobacterium pseudolongum*		Improvement of inflammation in HFD-fed mice.

(1) CMC, P80, soy lecithin, sunflower lecithin, maltodextrin, propylene glycol alginate, iota carrageenan, kappa carrageenan, lambda carrageenan, xantham gum, gum arabic, guar gum, locust bean gum, agar-agar, diacetyl tartaric acid ester of mono- and diglycerides, hydroxypropyl methylcellulose, sorbitan monostearate, mono- and diglycerides, glyceryl stearate, glyceryl oleate. Abbreviations: ADI, acceptable daily intake; CD, Crohn’s disease; CMC, carboxymethyl cellulose; DSS, dextran sulfate sodium; GF, germ-free; GLP1R, glucagon-like peptide 1 receptor; GLP2R, glucagon-like peptide 2 receptor; GML, glycerol monolaurate; HFD, high-fat diet; IBD, inflammatory bowel disease; IFN, IFN; IL, interleukin; LCN2, lipocalin 2; LPS, lipopolysaccharide; MPO, myeloperoxidase; SHIME, Simulator of the Human Intestinal Microbial Ecosystem; P80, polysorbate 80; RCT, randomized controlled trial; SCFA, short-chain fatty acid; TGF, transforming growth factor; TLR, Toll-like receptor; TNF, tumor necrosis factor; WT, wild type.

### 4.3. Food Colorants, Gut Microbiota, and IBD

Food colorants are food additives, which are added to food mainly to make up for color losses following exposure to light, air, and temperature variations to enhance naturally occurring colors and to add colors to foods that would otherwise be colorless or colored differently [114]. One of the main food additives used for its coloring properties is titanium dioxide (TiO_2_) coded as E171 [115]. In May 2021, the Panel of EFSA concluded that E171 can no longer be considered safe when used as a food additive [116]. TiO_2_ nanoparticles (NPs) were present in different kinds of products such as sauces, cheeses, skimmed milk, ice-cream, coating for sweets, and chewing gum [117]. Increased consumption of TiO_2_ NPs can negatively impact the human microbiome [118]. Several studies demonstrated changes in gut microbiota composition with an increase in *Firmicutes* [119,120]. Some studies in vitro have shown that TiO_2_ NPs and their aggregates can damage microvilli structure and alter epithelial integrity [121,122,123]. Furthermore, TiO_2_ NPs stimulate an increase in mucus production [119]. In vivo consumption of TiO_2_ can alter the composition and the activity of intestinal bacteria, promote an inflammatory environment in the gut, and aggravate gut barrier impairment and immune responses in animals already affected by diseases such as colitis, IBD, and obesity [115]. As regards other food colorants, a recent study showed that the commonly used food colorants azo dyes Red 40 and Yellow 6 can trigger IBD-like colitis in mice conditionally expressing IL-23 or in two additional animal models in which IL-23 expression was augmented [124]. These findings confirm the pivotal role of the cytokine IL-23 in the pathogenesis of IBD and colitis-associated colon cancer. Indeed, genetic studies revealed that subgroups of IBD patients have single-nucleotide polymorphisms in the IL-23R gene, suggesting that IL-23R signaling affects disease susceptibility [125].

### 4.4. Other Molecules Added to Food, Gut Microbiota, and IBD

#### 4.4.1. Maltodextrin

The polysaccharide MDX is often added during food production as a thickener, filler, or coating agent and has the ability to modulate gut microbiota [14]. MDX can expand the ileal resident population of *Escherichia coli*, inducing necrotizing colitis in piglets [126], and it can enhance the cellular adhesion of the adherent-invasive *Escherichia coli* strain [127], and it leads to a total increase in the bacterial load in the caecum of mice [128]. During the last decades, a concomitant rise in IBD incidence and MDX consumption was noted in the Western diet [129]. A possible link with IBD development and maintenance is sustained by the evidence that oral MDX consumption leads to aggravated gut inflammation [14]. Goblet cells of IBD-affected patients, in particular UC ones, contain fewer mucin granules filled with an altered Muc-2 precursor, which determine a very thin mucus layer due to the decreased production and secretion [130]. Moreover, MDX increases the endoplasmic reticulum stress in goblet cells [130]. The same cell aspect was discovered in mice orally fed with MDX [14]. Moreover, MDX associated with other sweeteners showed the capacity to promote the growth of *Bifidobacteria* in the human gut microbiota, probably due to the prebiotic role of MDX at the colonic level, even if this did not induced observable effects [82]. Thus MDX, in the contest of CD development, makes the gut more susceptible to epithelial damage due to the raised number of bacteria and impairs the natural host anti-microbial response [131].

#### 4.4.2. Food Preservatives

Preservatives are a group of agents added to prevent the natural deterioration of food due to microorganism growth. An in vitro study showed that sulfites can inhibit normal gut microbiota bacterial species [132]. Another preservative, ε-polylysine, can promote a change in mouse gut microbiota [133]. Some common human gut microbes with known beneficial anti-inflammatory effects (i.e., *Bacteroides coprocola*, *Clostridium tyrobutyricum*, *Lactobacillus paracasei*, etc.) are more sensitive to common anti-microbial preservatives than other microbes with pro-inflammatory colitogenic properties (i.e., *Enterococcus faecalis*, *Bacteroides thetaiotaomicron*, etc.) [134]. The same authors demonstrated how three very commonly used anti-microbial agents (sodium benzoate, E211; sodium nitrite, E250; potassium sorbate, E202) at normal exposure levels induce human gut microbial dysbiosis with a reduction in terms of diversity, the increase in the relative abundance of *Proteobacteria* phylum, and depletion of the *Clostridiales* order, which is frequently related to immune-mediated diseases [135].

#### 4.4.3. Aluminum

Aluminum (AL) use has increasing during recent years as an anticaking agent, defoaming agent, buffering agent, neutralizing agent, boiler compound, and emulsifier; moreover, it can be found in food if AL cooking utensils or packages are used [136]. The main contributors to AL exposure in adults are hot beverages and vegetables; children are exposed to AL mostly by vegetables, pasta, cake, and dairy-based desserts, while soy-based and ready-to-use milk products mainly contribute to exposure in infants [136]. Of the total AL bioavailability in humans, around 1% is ingested [137], but 99% of non-absorbed AL has potential effects at the level of the gut microbial barrier [136].

In CD’s vitro model, AL was able to stimulate chronic inflammation and granuloma formation, potentiating the expression of the pro-inflammatory cytokines [138]. Moreover, AL showed the capacity to enhance the inflammasome through the production and secretion of IL-1β, IL-18, and caspase 1 and by stimulating the IL-1 secretion from the human monocytes [139]. Other potential mechanisms involving AL in CD’s pathogenesis are the enhancing of reactive oxygen species generation, Th2 to Th1 shifting, and the altering of the membrane structure of dendritic cells [140].

#### 4.4.4. Nanoparticles

The use of nanoparticles (i.e., silver, silicon dioxide, iron oxide, zinc oxide, etc.) in food industries and agriculture has grown in recent years [141]. Silver induced (i) the reduction of up to 73% of *Faecalibacterium prausnitzii* [142] and (ii) the reduction in *Clostridium* species [143], which is also typical in IBD patients. Zinc oxide exposition was related to a global reduction in microbiota diversity [144], in particular *Firmicutes* [145]. On the contrary, silicon and titanium dioxide increased *Proteobacteria* [146]. In the study by Taylor et al., three nanoparticles (zinc oxide, titanium dioxide, and cerium dioxide) were administered at the environmentally relevant concentration in murine models, causing non-lethal but significant changes of gut microbial populations [147].

Given the growth of use of these nanoparticles in various industries, more robust studies are needed to deeply explore the relationship between those and IBD pathogenesis.

#### 4.4.5. Antioxidants

Antioxidants (i.e., phenolic acids, flavonoids, etc.) are natural compounds present in fruits, vegetables, spices, honey, etc. [148]. Hydroxycinnamic acids (caffeic acid, ferulic acid, sinapic acid, chlorogenic acid) are a major class of phenolic acids, and their interactions with gut microbiota are rapidly growing interest in research and have been tested on murine models to identify their possible role in colitis and IBD [149].

Natural antioxidants are added to meat-based products to avoid lipid oxidation [148]. In a study conducted on chronic inflammation-induced mice models, after several antioxidant preparations, a standardization in ileal and colic microbiota was found [148].

Caffeic acid showed the capacity to reduce the severity of bowel damage at histological and biochemical levels [150], suppressing inflammatory cytokines (i.e., TNF-α, IL-6, IFN-γ) secretion through the inactivation of NF-kB [149] to reduce the T-cells colonic infiltration, significantly alleviating colitis [151].

Supplementation of ferulic acid showed a reduction in colitis, maintaining T-helpers T1/T2 balance, inhibiting NF-κB, and differentiating regulatory T cells [152].

Sinapic acid reduces diarrhea in colitis-induced murine models, reducing TNF-α and myeloperoxidase production [153].

Chlorogenic acid seems to improve inflammation of colitis mice models, reducing NF-κB and myeloperoxidase [154], reducing oxidative stress and apoptosis markers [155], and lowering pro-inflammatory cytokines [156]. Moreover, some authors also observed an increase in microbial abundances, such as levels of *Akkermansia* and *Lactobacillus*, and a decrease in the pro-inflammatory bacterial species involved in IBD-like *Escherichia coli* and *Salmonella* spp. [157].

## 5. Conclusions

**Figure 2:**
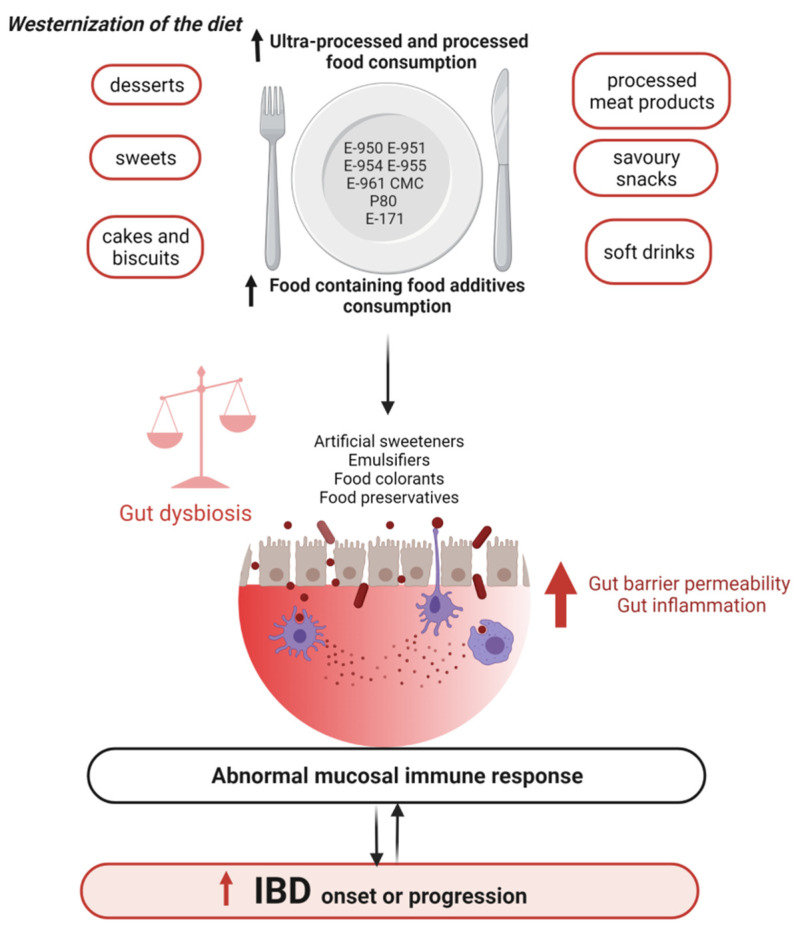
Food additives, gut microbiota, and IBD. Abbreviations: CMC, carboxymethyl cellulose; E-171, titanium dioxide; E-950, acesulfame potassium; E-951, aspartame; E-954, saccharin; E-955, sucralose, E-961, neotame; IBD, inflammatory bowel disease; P80, polysorbate 80.

Diet has a key role in the prevention and treatment of IBD. Many studies have demonstrated that the Western diet increases the risk of developing IBD. More recently, evidence has been accumulating about the severe impact of the consumption of several artificial food additives on gut microbiota and their positive associations with IBD through gut microbial modulation (Figure 2) [158].

This review highlighted that, although a wide range of food additives are approved by national authorities, some of them, such as artificial emulsifiers and artificial sweeteners, can negatively affect gut microbiota composition and functions (Figure 2). These gut microbial variations lead to the promotion of pro-inflammatory intestinal microbiota, disruption of mucus barrier, increased intestinal permeability, activation of inflammatory pathways such as LPS, flagellin, and pro-inflammatory cytokines production [159], exacerbating dysbiosis and mechanisms associated with IBD pathogenesis. In preclinical studies, such changes in bacteria triggered chronic colitis in mice genetically subject to this disorder, due to abnormal immune systems [95]. On the other hand, this review also showed that certain natural food additives such as stevia, soy/rapeseed lecithin, and natural antioxidants can improve gut microbiota composition and their functions.

These in vivo findings need to be confirmed in humans. Moreover, many confounders must be considered such as excessive high-dose exposure, which is irrelevant for human consumption, the sample size, or the background diet. Indeed, the synergies between different consumed processed food products, with or without the combination of other macronutrients, micronutrients, and fiber, also influence our gut microbiota. Investigating how food additive exposure affects the gut microbiota in IBD patients and healthy subjects has ethical issues; therefore, studies evaluating the impact of food additive consumption on the microbiota need to be conducted in relevant animal models with a large sample size or clinical populations at relevant exposure levels. In the future, preclinical studies could also assess the impact of the synergies of different food additives on the gut microbiota, varying their frequency of consumption, their type, or their artificial/natural origin [112].

It would be advisable to limit the human exposure to certain artificial food additives, reducing the consumption of ultra-processed foods, especially in patients with an IBD predisposition/diagnosis. We also recommended that IBD patients systematically undergo nutritional counseling by a clinical dietitian to follow a balanced and personalized diet prioritizing homemade food to control the amount of food additive exposure.

## Figures and Tables

**Table 1:** Effects of artificial sweeteners on gut microbiota and immune/inflammatory system in animal and human model studies.

First Author, Year	Artificial Sweeteners	Model	Main Results		
			Impact on Microbiota Composition	Impact on the Gut Barrier and Intestinal Permeability and SCFAs Synthesis	Impact on the Immune and Inflammatory System
Ahmad, 2020 [84]	Standardized (dose of 14% of the ADI for aspartame and 20% of the ADI for sucralose	RCT of healthy volunteers (duration of treatment 14 days)	Minimal effect on gut microbiota composition	Minimal effect on SCFAs production	
Bian, 2017a [81]	Sucralose	Mice			Increased expression of bacterial pro-inflammatory mediators: LPS, flagella protein synthesis, fimbriae, Shiga toxin
Bian, 2017a [83]	Saccharin	Mice			Increased inflammation factors; iNOS enzyme, TNFα in the liver. Increased expression of LPS, flagellar assembly, and bacterial toxins. Increased pro-inflammatory metabolites
Escoto, 2021 [76]	Sucrose Sucralose Stevia	Mice	Sucrose and sucralose: Reduction in bacterial communities Stevia: Improvement in bacterial diversity		Sucrose and sucralose: Increase in CD19+, a decrease in IgA+ and TGF-b, and an increase in IL-12 and IL-17 in Peyer’s patches Stevia: Increased percentage of CD19+ lymphocytes Minimal increase in IgA+, TGF-b, and IL-12 Decrease in IL-17
Farid 2020 [92]	Sucrose, Splenda^®^ or stevia	Mice	Reduced gut microbiota diversity		Increased pro-inflammatory cytokines (IL6, IL8) Reduction in anti-inflammatory cytokine IL10 in mice fed sucralose or stevia
Gerasimidis, 2020 [82]	Aspartame-based sweetener, sucralose, stevia	Human microbiota	Stevia: Microbial Shannon α-diversity increased Sucralose: Increased abundance of *Escherichia/Shigella and Bilophila*	Aspartame: Increased propionate and acetate production	
Guo, 2021 [77]	Sucralose	Mice	Intestinal microbiota changes	Alteration of gut barrier	Exacerbation in the severity of colitis Increased expression of pro-inflammatory cytokines Activation of the TLR5-MyD88-NF-κB signaling pathway
Hanawa, 2021 [72]	Acesulfame potassium	Mice	Induced microbial changes	Induced intestinal injury with enhanced lymphocyte migration to the intestinal mucosa Increased intestinal permeability Decreased expression of GLP-1R and GLP-2R	Increased expression of pro-inflammatory cytokines
Li, 2020 [75]	Sucralose	Mice	Intestinal dysbiosis	Decreased gut barrier integrity.	Increased tumorigenesis and worsening of DSS severity Decreased beta glucuronidase
Chi, 2018 [80]	Neotame	Mice	Reduction in the alpha-diversity Alteration the beta-diversity	Decrease in expression of butyrate synthetic genes	
Rosalez-Gomez, 2018 [78]	Sucrose, Splenda^®^ or stevia	Mice	Intestinal dysbiosis		Splenda^®^ and Stevia: Increased percentage of lymphocytes and IL6 and IL17 in Peyer’s patches and lamina propria Sucralose and stevia Increased leptin and C-peptide
Palmas,2014 [79]	Aspartame	Rats	Increased total bacteria abundance Increased abundance of *Enterobacteriaceae* and *Clostridium leptum* Attenuation of the typical high fat-induced increase in the Firmicutes: *Bacteroidetes* ratio		
Rodriguez- Palacios 2018 [74]	Sucralose	Mice	Intestinal dysbiosis (Proteobacteria expansion)		Increased inflammation (MPO activity)
Sanchez- Tapia 2020 [86]	Sucralose, steviol glycosides, or sucrose	Mice		Increased expression of bacterial genes involved in the synthesis of SCFAs	Sucralose: Increased pro-inflammatory cytokines and bacterial genes involved in the synthesis of LPS
Serrano, 2021 [85]	Saccharin, lactisole, or saccharin	RCT of healthy volunteers (duration of treatment 10 weeks) and mice model	No alteration of gut microbiota composition	No variations of SCFAs production	
Uebanso, 2017 [73]	Sucralose Acesulfame-potassium	Mice	Sucralose: Reduced amount of *Clostridium cluster XIVa*		
Wang, 2019 [87]	Sucralose	Mice		Increased gut damage Increased permeability	Increased inflammation (MPO, TNFalpha, and IL1b) Increased digestive proteases fecal chymotrypsin and trypsin Decreased beta-glucuronidase

Abbreviations: ADI, acceptable daily intake; DSS, dextran sodium sulfate; GLP-1R, glucagon-like peptide-1 receptor; NOS, nitric oxide synthase; IL, interleukin; LPS, lipopolysaccharide; MPO, myeloperoxidase; RCT, randomized controlled trial; SCFA, short-chain fatty acid; TLR, Toll-like receptor; TNF, tumor necrosis factor.

## Supplementary Materials

The following are available online at https://www.mdpi.com/article/10.3390/microorganisms10010167/s1, Table S1: Search strategy performed on 27 October 2021.
