# Supplementary material for: Food Additives, a Key Environmental Factor in the Development of IBD through Gut Dysbiosis
Source: Microorganisms. 2022 Jan 13;10(1):167. doi: 10.3390/microorganisms10010167 (PMC8780106; doi:10.3390/microorganisms10010167)
Supplement: Supplementary file 1 [file microorganisms-10-00167-s001.zip › microorganisms-1533744-supplementary.pdf]

Table S1. Search strategy performed on 27th October 2021

**MEDLINE**	**Search terms**	**Results**
**#1**	ibd OR (Inflammatory AND bowel AND disease*) OR (Crohn* AND disease*) OR (ulcerative* AND colitis*)	137,251
**#2**	microbiota OR (gut AND microbiota) OR microbiome OR (intestinal AND flora)	110,750
**#3**	**(**Artificial AND food AND additive*) OR (Artificial AND sweeteners) OR (Polyol*) OR (Acesulfame AND potassium) OR Aspartame OR Saccharin OR Sucralose OR Cyclamate OR Neotame OR Emulsifier* OR (Carboxymethyl AND cellulose) OR (Polysorbate AND 80) OR (Food AND color*)OR (Food AND preservative*) OR (Benzoic AND acid) OR (Sodium AND benzoate) OR (Titanium AND dioxide) OR (Sodium AND nitrite) OR (Food AND additive*)	151,005
**#1 AND #2 AND #3**		**276**
**WEB OF SCIENCE**	**Search terms**	**Results**
**#1**	ibd OR (Inflammatory AND bowel AND disease*) OR (Crohn* AND disease*) OR (ulcerative* AND colitis*)	173,061
**#2**	microbiota OR (gut AND microbiota) OR microbiome OR (intestinal AND flora)	129,185
**#3**	**(**Artificial AND food AND additive*) OR (Artificial AND sweeteners) OR (Polyol*) OR (Acesulfame AND potassium) OR Aspartame OR Saccharin OR Sucralose OR Cyclamate OR Neotame OR Emulsifier* OR (Carboxymethyl AND cellulose) OR (Polysorbate AND 80) OR (Food AND color*)OR (Food AND preservative*) OR (Benzoic AND acid) OR (Sodium AND benzoate) OR (Titanium AND dioxide) OR (Sodium AND nitrite) OR (Food AND additive*)	274,716
**#1 AND #2 AND #3**		**307**
**SCOPUS**	**Search terms**	**Results**
**#1**	ibd OR (Inflammatory AND bowel AND disease*) OR (Crohn* AND disease*) OR (ulcerative* AND colitis*)	168,821
**#2**	microbiota OR (gut AND microbiota) OR microbiome OR (intestinal AND flora)	126,655
**#3**	(Artificial AND food AND additive*) OR (Artificial AND sweeteners) OR (Polyol*) OR (Acesulfame AND potassium) OR Aspartame OR Saccharin OR Sucralose OR Cyclamate OR Neotame OR Emulsifier* OR (Carboxymethyl AND cellulose) OR (Polysorbate AND 80) OR (Food AND color*)OR (Food AND preservative*) OR (Benzoic AND acid) OR (Sodium AND benzoate) OR (Titanium AND dioxide) OR (Sodium AND nitrite) OR (Food AND additive*)	435,388
**#1 AND #2 AND #3**		**268**
